# Supplementary figures and images for: Client Applications and Server-Side Docker for Management of RNASeq and/or VariantSeq Workflows and Pipelines of the GPRO Suite
Source: Genes (Basel). 2023 Jan 19;14(2):267. doi: 10.3390/genes14020267 (PMC9957322; doi:10.3390/genes14020267)

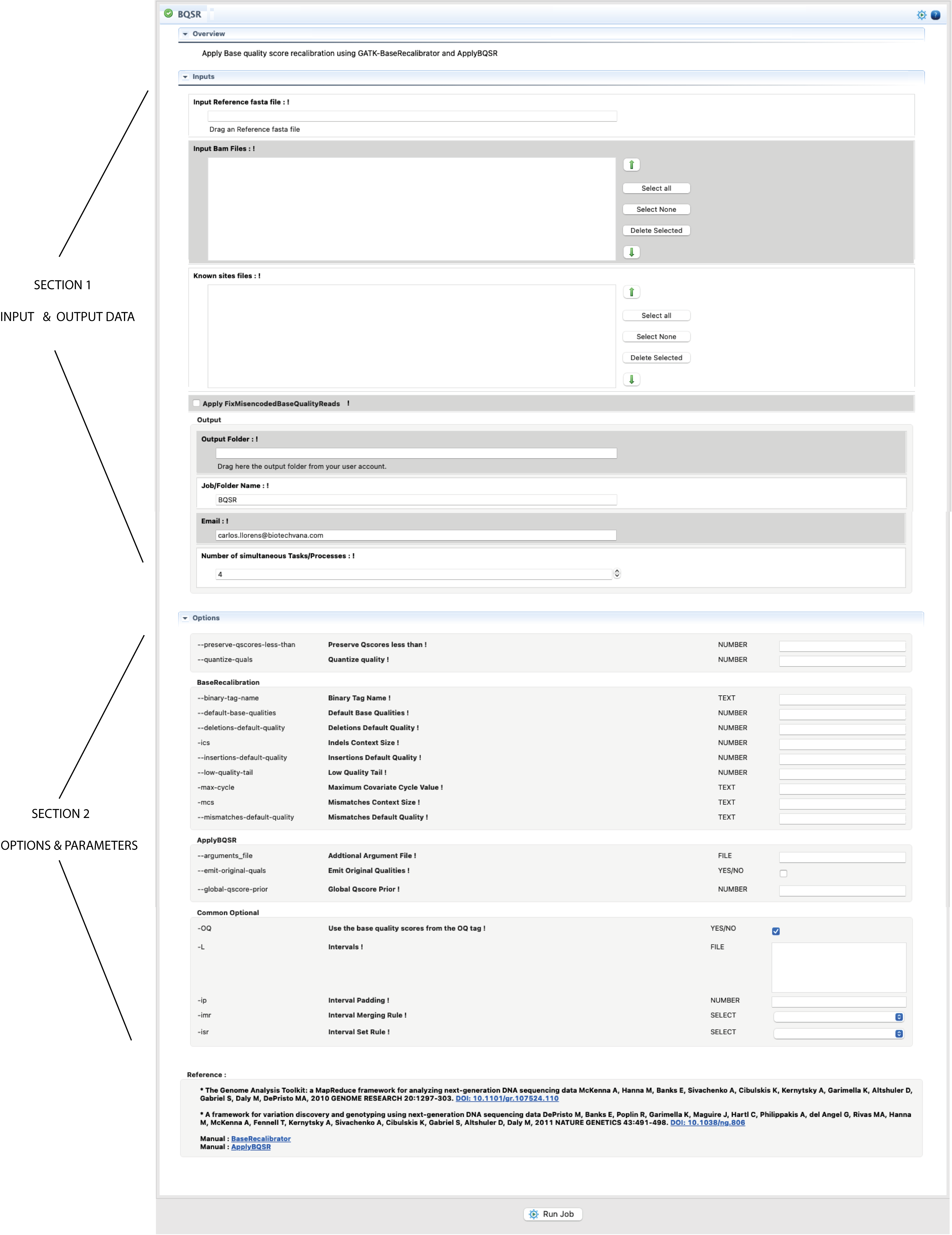

Supplement: Supplementary file 1 [file genes-14-00267-s001.zip › Supplementary_files/Supplementary_file_S3.png]

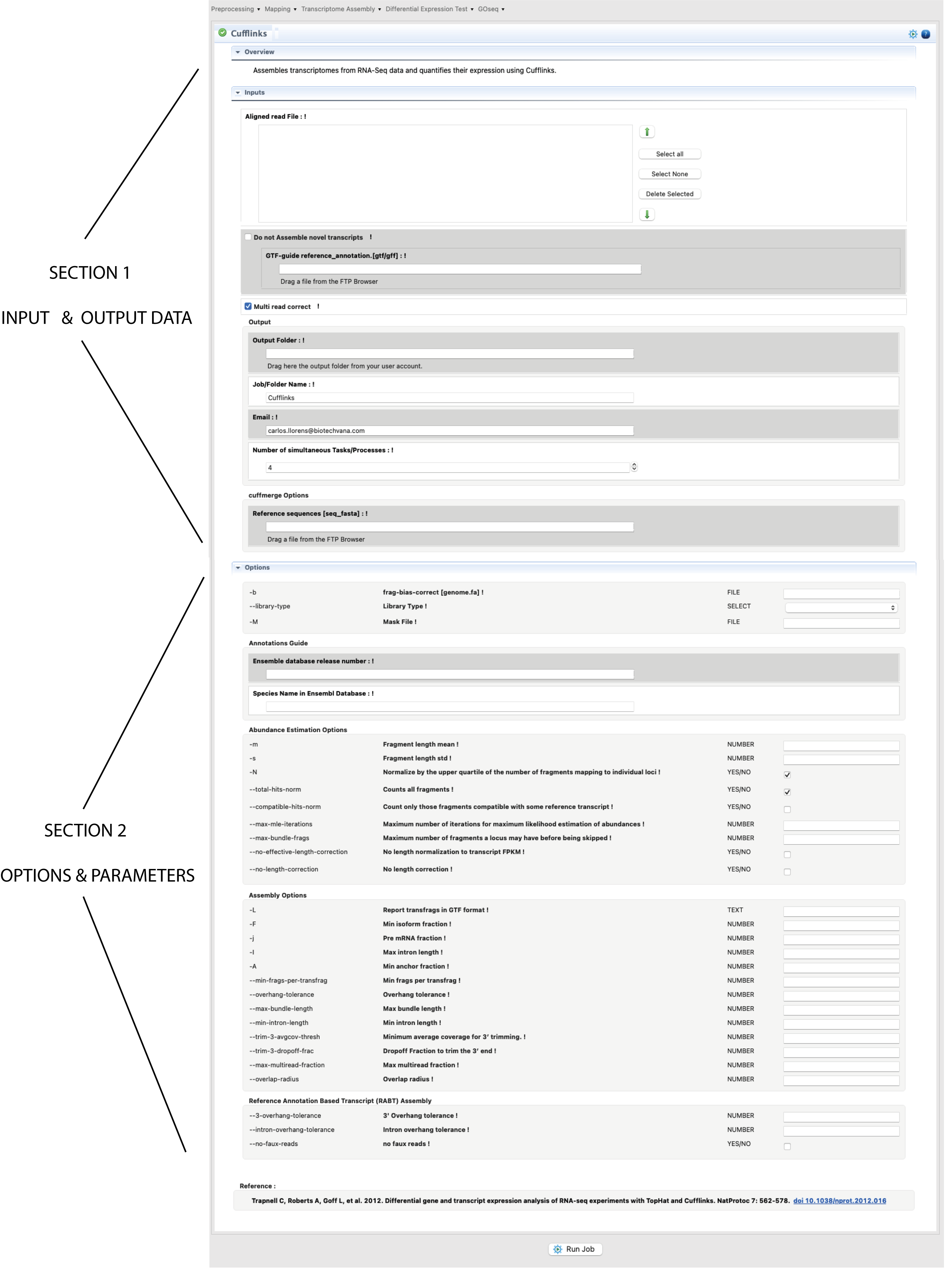

Supplement: Supplementary file 1 [file genes-14-00267-s001.zip › Supplementary_files/Supplementary_file_S2.png]

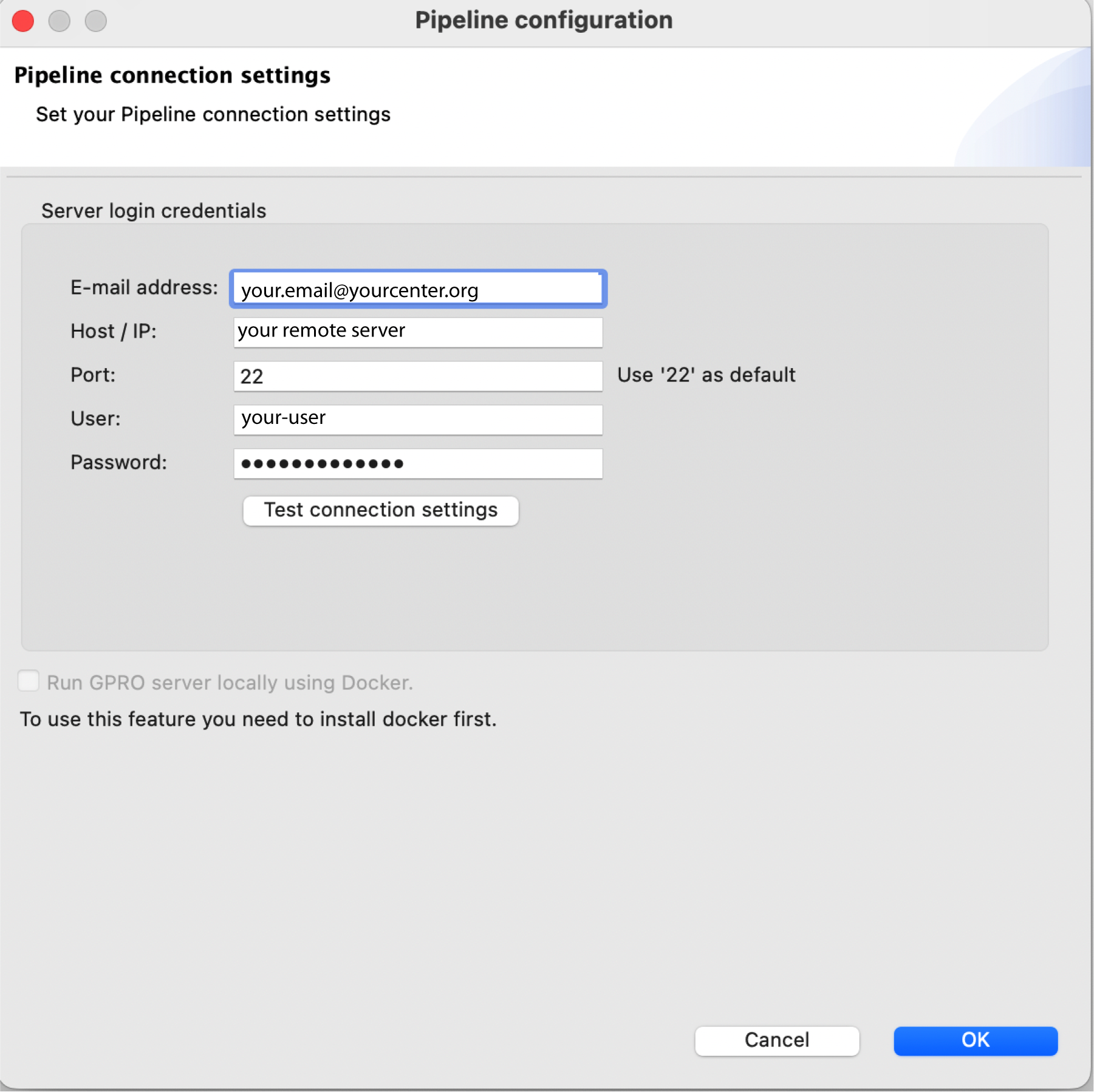

Supplement: Supplementary file 1 [file genes-14-00267-s001.zip › Supplementary_files/Supplementary_file_S1.png]

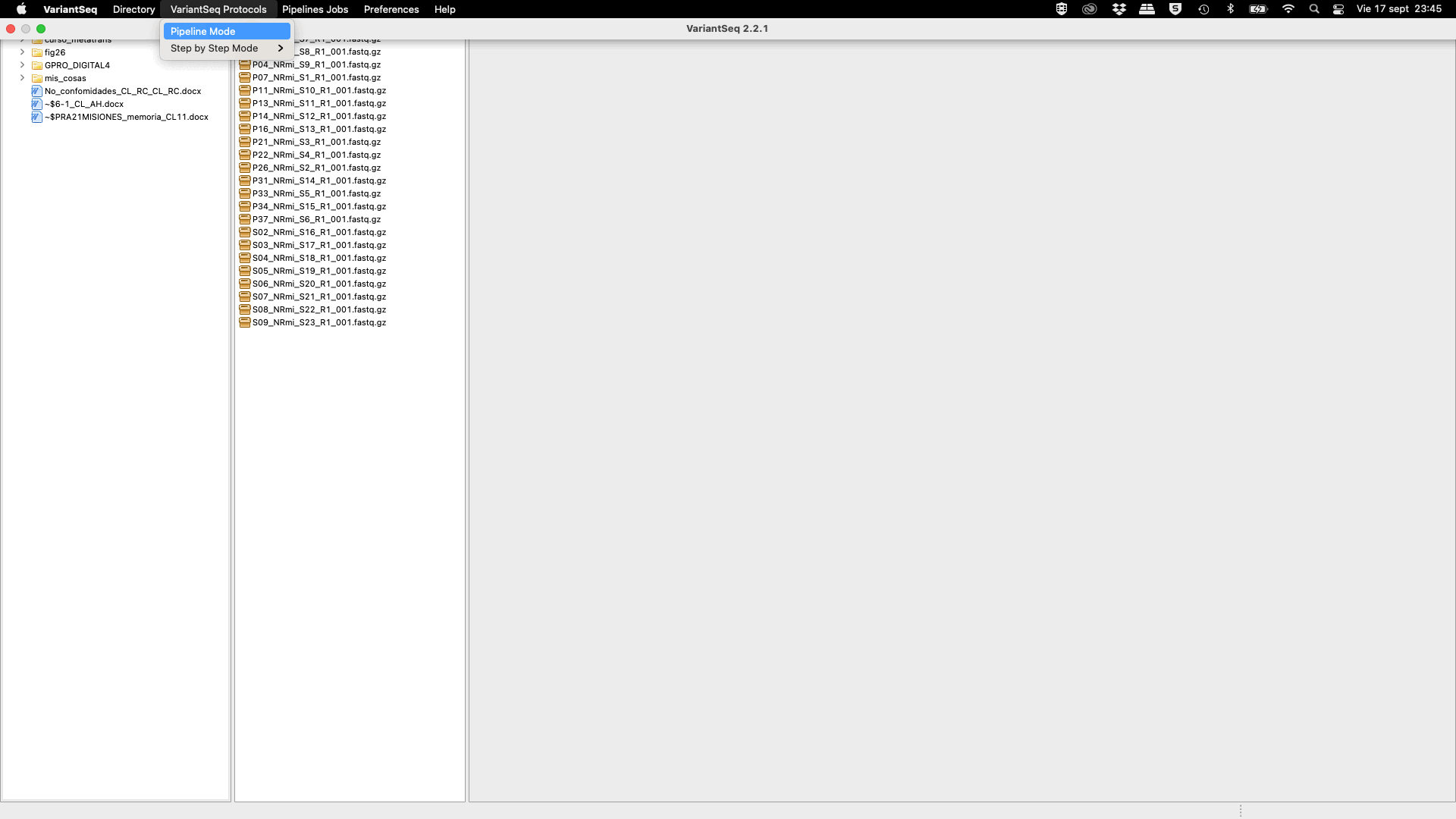

Supplement: Supplementary file 1 [file genes-14-00267-s001.zip › Supplementary_files/Supplementary_file_S5.gif]

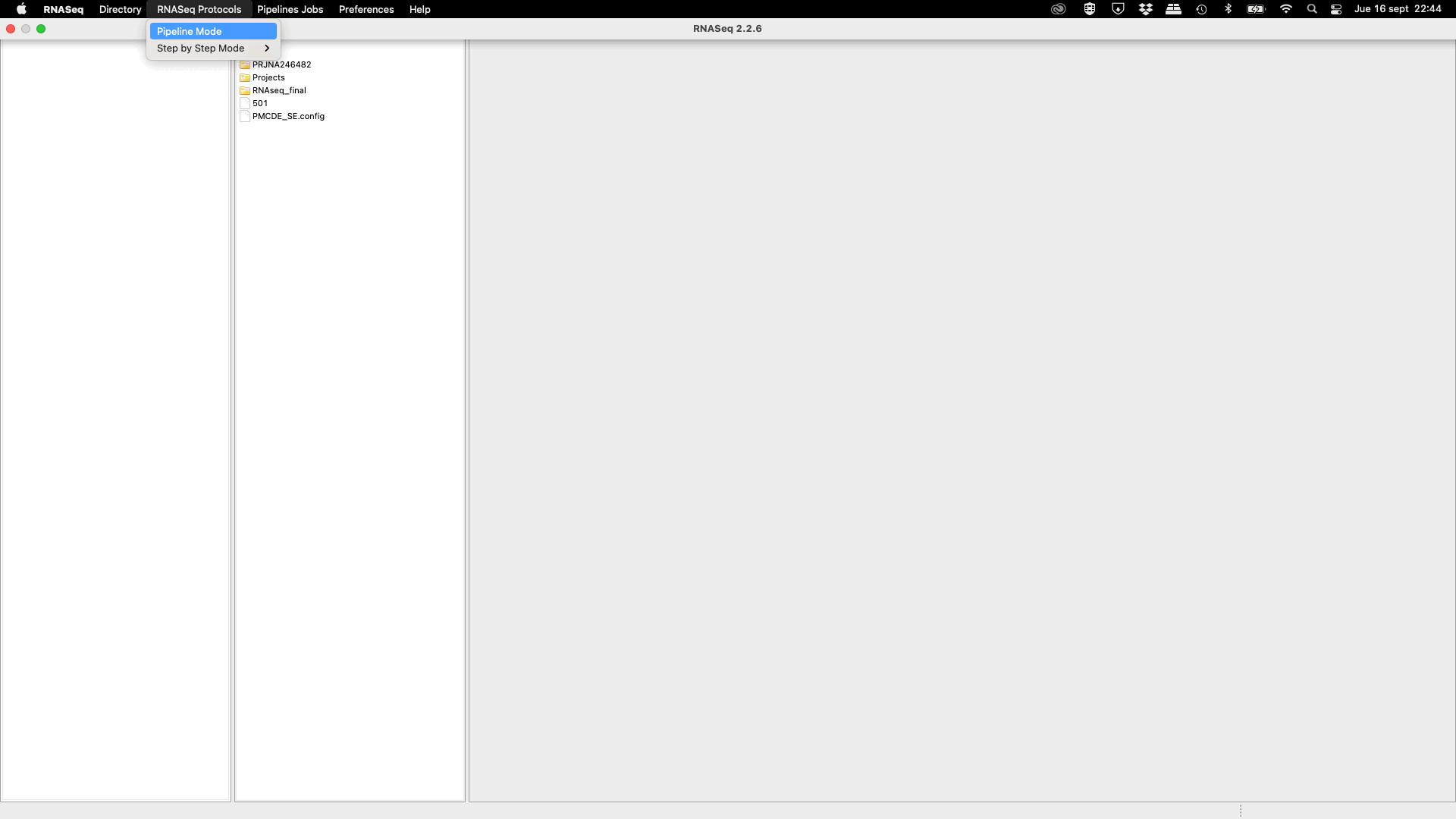

Supplement: Supplementary file 1 [file genes-14-00267-s001.zip › Supplementary_files/Supplementary_file_S4.gif]
